# Tigecycline challenge triggers sRNA production in *Salmonella enterica* serovar Typhimurium

**DOI:** 10.1186/1471-2180-12-195

**Published:** 2012-09-07

**Authors:** Jing Yu, Thamarai Schneiders

**Affiliations:** 1 Centre for Infection and Immunity, Medical Biology Centre, Queen’s University Belfast, 97 Lisburn Road, Belfast, BT9 7BL, UK

## Abstract

**Background:**

Bacteria employ complex transcriptional networks involving multiple genes in response to stress, which is not limited to gene and protein networks but now includes small RNAs (sRNAs). These regulatory RNA molecules are increasingly shown to be able to initiate regulatory cascades and modulate the expression of multiple genes that are involved in or required for survival under environmental challenge. Despite mounting evidence for the importance of sRNAs in stress response, their role upon antibiotic exposure remains unknown. In this study, we sought to determine firstly, whether differential expression of sRNAs occurs upon antibiotic exposure and secondly, whether these sRNAs could be attributed to microbial tolerance to antibiotics.

**Results:**

A small scale sRNA cloning strategy of *Salmonella enterica* serovar Typhimurium SL1344 challenged with half the minimal inhibitory concentration of tigecycline identified four sRNAs (sYJ5, sYJ20, sYJ75 and sYJ118) which were reproducibly upregulated in the presence of either tigecycline or tetracycline. The coding sequences of the four sRNAs were found to be conserved across a number of species. Genome analysis found that sYJ5 and sYJ118 mapped between the 16S and 23S rRNA encoding genes. sYJ20 (also known as SroA) is encoded upstream of the *tbpAyabKyabJ* operon and is classed as a riboswitch, whilst its role in antibiotic stress-response appears independent of its riboswitch function. sYJ75 is encoded between genes that are involved in enterobactin transport and metabolism. Additionally we find that the genetic deletion of sYJ20 rendered a reduced viability phenotype in the presence of tigecycline, which was recovered when complemented. The upregulation of some of these sRNAs were also observed when *S.* Typhimurium was challenged by ampicillin (sYJ5, 75 and 118); or when *Klebsiella pneumoniae* was challenged by tigecycline (sYJ20 and 118).

**Conclusions:**

Small RNAs are overexpressed as a result of antibiotic exposure in *S.* Typhimurium where the same molecules are upregulated in a related species or after exposure to different antibiotics. sYJ20, a riboswitch, appears to possess a *trans*-regulatory sRNA role in antibiotic tolerance. These findings imply that the sRNA mediated response is a component of the bacterial response to antibiotic challenge.

## Background

Multiple studies demonstrate that non coding RNAs (or small RNAs (sRNAs)) possess regulatory roles in the bacterial stress response [1-4]. Bacterial sRNA regulators typically range from 50 – 250 nts and are often transcribed from intergenic regions (IGRs), although open reading frames may also encode sRNAs [5]. Most sRNAs act as regulators at the post-transcriptional level by base-pairing with target mRNAs; these sRNA-mRNA binding regions are often short and imperfect and may require an additional RNA chaperone, which in most cases is the Hfq protein [6,7]. This imperfect binding allows each sRNA molecule to control multiple targets [8], where either the translation of the target mRNA is upregulated, or more commonly inhibited.

Many sRNA regulators are upregulated when bacteria sense environmental stress: these include oxidative stress [1], low pH environment [2], nutrient deprivation [4] and glucose-phosphate stress [3]. Despite overwhelming evidence that sRNAs play a role when bacteria experience physiological stress, no systematic study has been undertaken to ascertain the impact or levels of sRNA production in bacteria when antibiotics are present.

Naturally susceptible pathogens can develop drug resistance when treated with antibiotics [9]. Genetically acquired antibiotic resistance in pathogenic bacteria, via spontaneous / random mutations and horizontal gene transfer, is a significant issue in the treatment of infectious diseases [10]. Intrinsic regulatory networks such as those mediated by the transcriptional regulators MarA, SoxS and RamA are also implicated in the development of antibiotic resistance particularly since these systems control the influx / efflux of antibiotics [11]. Thus far studies that have focused on the intrinsic antibiotic resistome are limited to gene and protein networks mediated by these gene operons or other transcription factors [11-13]. Hence the role of the newly uncovered class of regulatory molecules such as sRNAs in controlling or contributing to the antimicrobial resistance phenotype is largely unknown. Some evidence for the role of sRNAs in mediating antimicrobial resistance already exists: for example, the expression of bacterial outer membrane proteins, OmpF and OmpC, involved in antibiotic import, is controlled by the sRNAs MicF and MicC respectively [14-16]. Additionally, the overexpression of another sRNA (DsrA) was recently found to induce multidrug resistance in *Escherichia coli* via the MdtEF efflux pump [17]. Nevertheless, whether the functional role of MicF, MicC and DsrA is indeed part of the bacteria’s intrinsic stress response to antibiotic challenge remains unknown.

Tigecycline is a member of the glycylcycline group of antibiotics, and was registered in the EU in April 2006 [18]. This bacteriostatic antibiotic acts as a protein synthesis inhibitor by binding to the 30S ribosomal subunit [19]. Tigecycline is active against a broad range of bacteria, with only few naturally resistant exceptions, namely, *Proteus spp*., *Morganella morganii*, *Providencia spp*., and *Pseudomonas aeruginosa*. Specifically, tigecycline is effective against multidrug resistant bacteria such as *Staphylococcus aureus* (MRSA), vancomycin-resistant *Enterococcus* (VRE), extended-spectrum beta-lactamase (ESBL)-expressing Enterobacteriaceae, and carbapenem-resistant strains [20-22]. Reports of resistance to tigecycline have been rare in naturally susceptible pathogens, however in resistant variants efflux pump overexpression has contributed to tigecycline resistance [23-28].

*Salmonella*, a member of Enterobacteriaceae, encodes both the *ramA* transcriptional factor and the *acrAB* efflux pump, which when overexpressed confers tigecycline resistance [29]. Additionally, *Salmonella* represents a model bacterium for sRNA mining [30] and genome manipulation [29], making it an ideal system for our study, but more importantly represents a paradigm for other members of Enterobacteriaceae. Hence in this study we used a cloning strategy to determine the sRNA population after tigecycline exposure in *Salmonella enterica* serovar Typhimurium, and also whether the absence of these sRNAs would render the cells less adaptable to tigecycline challenge.

## Results

### cDNA library construction and analysis

**Figure 1:**
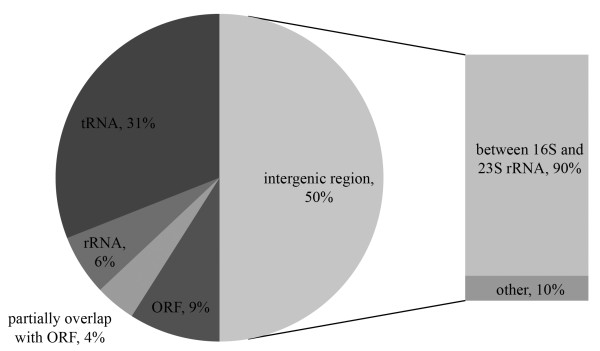
**A pie chart representation illustrating the cDNA sequences distribution pattern on the SL1344 chromosome.** The results showed that 50% of the sequences are encoded within IGRs, 90% of which are situated between 16S and 23S rRNA (shown on the right), 31% are tRNA sequences, 6% are part of rRNA sequences, 9% completely overlap with ORFs, and 4% partially overlap with ORFs.

A cDNA library was constructed from the cells that were challenged by half the minimal inhibitory concentration (MIC) of tigecycline (0.125 μg/ml) at OD_600_ = 0.6. Approximately ~6000 clones were obtained; from these 200 random candidates were sequenced and analysed. The nature of the cDNA library construction procedure (see Materials and Methods) allowed us to obtain the sequences in an orientation specific manner. The cDNA sequences were mapped to the *S.* Typhimurium SL1344 genome (FQ312003) using BLAST ( http://blast.ncbi.nlm.nih.gov/Blast.cgi). Of the mapped sequences, 31% encoded tRNAs; 6% and 9% matched to rRNAs and protein coding sequences, respectively; 4% partially overlapped with open reading frames (ORFs), and 50% aligned to IGRs. Of all the IGR readings, 90% were located between the 16S and 23S rRNA encoding genes (Figure 1).

Analyses of the cDNA sequences encoding partial ORFs indicated which genes were expressed in the presence of tigecycline. As stated above, 9% of the sequences identified matched to rRNAs, in addition to a further sequence which was found to overlap the 30S ribosomal protein and another mapped to elongation factor *tu*. This is perhaps not surprising, given that the specific target for tigecycline is the ribosome [19]. On the other hand, sequences overlapping known stress-response genes were also captured in the cDNA library, e.g. *dinF* and a gene encoding a putative outer membrane protein (SL1344_1151). The *dinF* gene is a member of the SOS response family and encodes an efflux pump which belongs to the multidrug and toxic compound extrusion (MATE) family [31], and SL1344_1151, encoding a putative outer membrane protein homologous to *ycfR* in *E. coli*, which influences biofilm formation through stress response and surface hydrophobicity [32]. The expression of these genes supports our hypothesis that challenge at half the MIC of tigecycline triggers a stress response. Of note, the cDNA library also contained sequences of different lengths that mapped to open reading frames, which we postulate to be a result of mRNA degradation, rather than a representation of *bona fide* sRNA regulators. Meanwhile, 4% of all sequences that partially overlap ORFs, all do so at the 5’ end of the ORFs. This suggests that these sequences might be 5’ untranslated regions, or encode riboswitches and/or control the expression of the downstream genes.

### Northern blot verification

**Figure 2:**
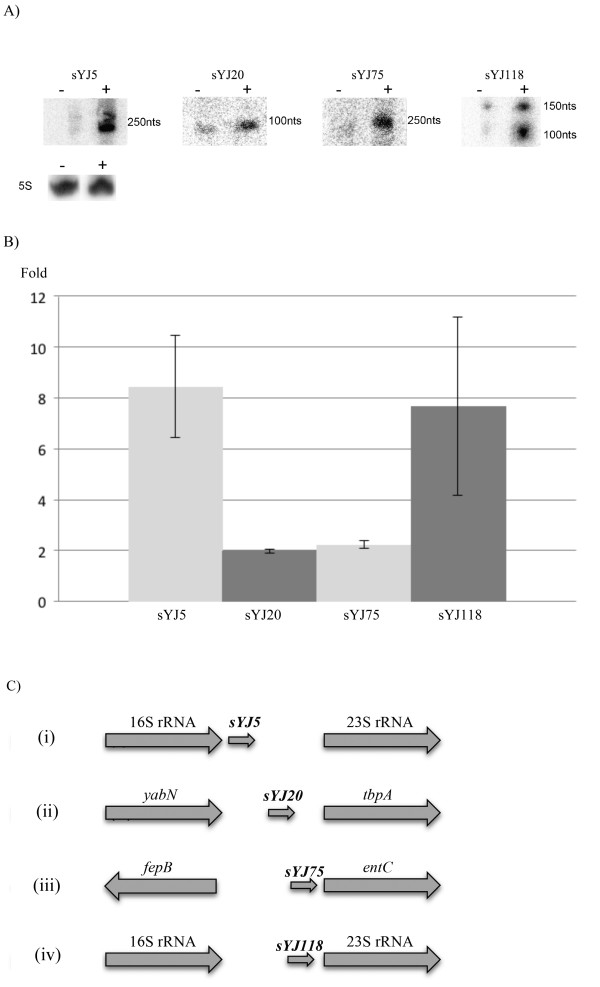
**(A) Northern blot analysis for the four sRNAs (sYJ5, sYJ20 (SroA), sYJ75 and sYJ118) that were upregulated in the presence of tigecycline, and (B) bar chart illustration of the overexpressed sRNAs and (C) chromosomal locations and the directions of transcription of sYJ5, sYJ20, sYJ75 and sYJ118.A)** Northern blot analysis for sYJ5, 20, 75 and 118. Image on top: all lanes marked by - were loaded with SL1344 total RNA extracted from cells grown under normal conditions (RDM, shaking, 37°C); all lanes marked by + were loaded with SL1344 total RNA extracted from cells challenged with half the MIC of tigecycline (0.125 μg/ml). Image below: representative image of the internal reference of 5S RNA levels in the same RNA samples. **B)** Densitometric analysis of the data from northern blot experiments of challenged / unchallenged cells with half the MIC of tigecycline. After normalisation to the 5S RNA levels, relative fold increases for sYJ5, 20, 75 and 118 were found to be 8, 2, 2, and 8 fold, respectively compared to unchallenged cells. Error bars are generated based on three independent experiments. **C)** The three coding sequences of sYJ5 are located in (1) SL1344_rRNA0001-rRNA0002, (2) SL1344_rRNA0014-rRNA0015 and (3) SL1344_rRNA0017-rRNA0018. The two identical copies of sYJ118 are encoded in (1) SL1344_rRNA0010-rRNA0009 and (2) SL1344_rRNA0011-rRNA0012, and the other five paralogs are found in (1) SL1344_rRNA0001-rRNA0002, (2) SL1344_rRNA0006-rRNA0005, (3) SL1344_rRNA0014-rRNA0015, (4) SL1344_rRNA0017-rRNA0018 and (5) SL1344_rRNA0020-rRNA0021.

**Figure 3:**
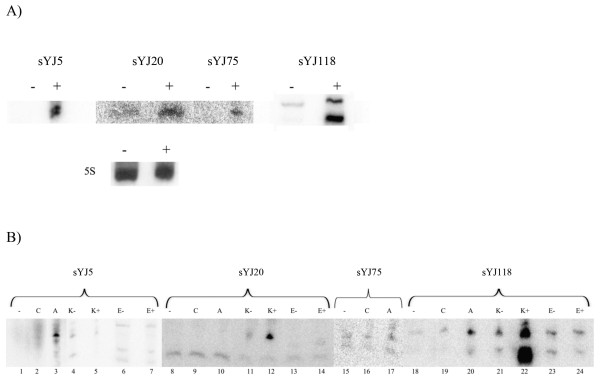
**Northern blots for sYJ5, sYJ20 (SroA), sYJ75 and sYJ118 A) in SL1344 challenged with half the MIC of tetracycline, B) ciprofloxacin or ampicillin, and the four sRNAs level in *E. coli *and *K. pneumoniae *challenged with half the MIC of tigecycline.A)** Lanes with - were loaded with control samples; lanes with + were loaded with total RNA extracted from cells challenged with half the MIC of tetracycline. This image is composite from different experiments. **B)** Lanes marked by - were loaded with control total RNA extracted from *S. Typhimurium*. Lanes marked as C were loaded with the total RNA extracted from *S. Typhimurium* that was challenged with half the MIC of ampicillin (1 µg/ml). Lanes marked by K- were loaded with the control total RNA extracted from *K. pneumoniae*. Lanes marked as K + were loaded with the total RNA extracted from *K. pneumoniae* that was challenged with half the MIC of tigecycline. Lanes marked as E- were loaded with the control total RNA extracted from *E. coli*. Lanes marked as E + were loaded with the total RNA extracted from *E. coli* that was challenged with half the MIC of tigecycline. Probe sequences were checked for 100% identity match in *K. pneumoniae* and *E. coli* prior to use.

**Figure 4:**
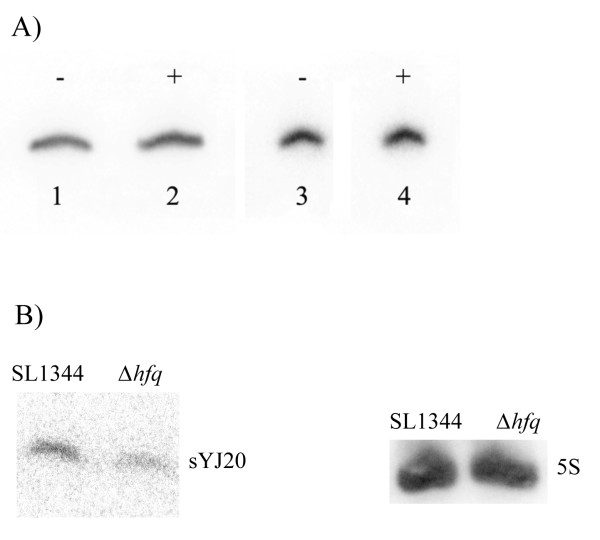
**Northern blots for A) the 5S RNA level in SL1344 and B) sYJ20 level in SL1344 and the Δ*hfq*strain (JVS-0255) in the presence of ciprofloxacin.A)** Lane 1 and 3 (also labelled as -) were loaded with SL1344 total RNA extracted from cells grown under normal conditions (RDM, shaking, 37°C); lane 2 was loaded with SL1344 total RNA extracted from cells challenged with half the MIC of tigecycline (0.125 μg/ml); lane 4 was loaded with SL1344 total RNFA extracted from cells challenged with half the MIC of tetracycline (1 μg/ml). All lanes were loaded with 125 ng of total RNA. The experiment was repeated 4 times. Densitometric analysis of the results showed little or no difference in 5S RNA expression level in the three growing conditions (5S_tigecycline_: 5S_control_ = 0.88, 5S_tetracycline_ : 5S_control_ = 1.15, average of 4 different experiments). **B)** Both strains (SL1344 and the *hfq* deletion strain (JVS-0255, Table 2)) were challenged with sub-inhibitory concentration of ciprofloxacin (0.0078 μg/ml) before the total RNA was extracted and probed for sYJ20 by northern blot. As shown above, the Δ*hfq* strain (right lane) produced less sYJ20 compared to SL1344 (left lane). 5S RNA was used as a loading control.

Northern blot analysis was performed on RNA extracted from SL1344 that were either unchallenged or challenged with half the MIC of tigecycline. Since most sRNAs are produced from IGRs [30], only sequences from these regions (100 out of 200 in total) were selected for further validation by northern blot analysis. As 90% of the IGR sequences are located between 16S and 23S rRNA coding sequences, most of which are identical, there were 20 unique IGR sequences (including those located between 16S and 23S rRNA) that were assayed, of which four (encoding sYJ5, sYJ20, sYJ75 and sYJ118) were found to consistently show elevated expression with tigecycline challenge (Figure 2A). The remaining sRNA candidates were either not detectable by northern blots, or did not show differential levels of transcription. Correspondingly all further analyses focused on these four sRNAs. The relative fold increase in sRNA expression was determined by northern blots in challenged versus unchallenged cells. Upon tigecycline exposure, the expression levels of sYJ5, sYJ20, sYJ75 and sYJ118 (performed in triplicate using densitometric analyses), relative to unchallenged cells, were increased to 8, 2, 2 and 8 fold, respectively (Figure 2A and B). We also tested the level of the four sRNAs in cells challenged with half the MIC of tetracycline (1 μg/ml). As expected, all of the four sRNAs were also found to be upregulated compared to the control sample (Figure 3A). This is possibly due to the fact that tigecycline and tetracycline are related compounds, and they may as well trigger stress response pathways that share a common set of regulatory molecules. Of note and as shown in Figure 4A, the level of 5S RNA was not affected by the presence of half the MIC of tigecycline or tetracycline (5S_tigecycline_: 5S_control_ = 0.88, 5S_tetracycline_ : 5S_control_ = 1.15, average of 4 different experiments).

### Bioinformatic analysis

All four sRNA sequences were searched against *S.* Typhimurium SL1344 using NCBI BLAST. The sYJ5 encoding sequence is located between the 16S (SL1344_rRNA0001) and 23S rRNA (SL1344_rRNA0002) coding loci on the sense strand (Figure 2C (i)). BLAST analysis uncovered two additional identical copies in the genome sequence of SL1344 (one between SL1344_rRNA0014 and SL1344_rRNA0015, the other SL1344_rRNA0017 and SL1344_rRNA0018).

Similar to sYJ5, sYJ118 is also encoded from the IGR between the 16S and 23S rRNA coding sequences, but from a different genetic locus (SL1344_rRNA0009 – SL1344_rRNA0010, Figure 2C (iv)). The sequence encoding sYJ118 has an identical copy (SL1344_rRNA0011 – SL1344_rRNA0012) and additionally five other paralogs with 93% - 99% identity on the SL1344 chromosome.

The encoding sequence of sYJ75 is flanked by *entC* downstream (encoding isochorismate synthase), and *fepB* upstream (encoding the iron-enterobactin transporter periplasmic binding protein) (Figure 2C (iii)). It also has a paralog that shares 90% identity, starting at position 1515629 on the *S.* Typhimurium SL1344 genome and located between *pntB* (encoding pyridine nucleotide transhydrogenase β subunit) and an un-annotated gene (encoding a putative membrane protein).

sYJ20 was previously identified by Vogel *et al.* in *E. coli* as SroA [5], encoded by a sequence downstream of *yabN* (encoding SgrR, a transcriptional regulator in *E. coli*[33]) and upstream of *tbpA* (encoding the thiamine-binding periplasmic protein, homologous to *thiB* in *E. coli*) (Figures 2C (ii) and 5A).

**Figure 5:**
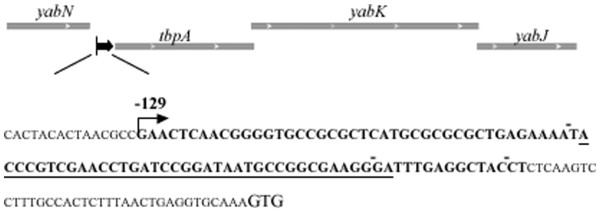
**The chromosomal location of the sYJ20 (SroA) encoding region and its encoding sequence.** sYJ20 is encoded upstream of the *tbpA-yabK-yabJ* operon, and the shared TSS of sYJ20 and *tbpA* as determined by 5’ RACE analysis is represented by the dark-black arrow. The DNA sequence of sYJ20 (SroA) is shown in bold letters, which is also the region that was deleted in YJ104 and used for TargetRNA prediction (Table 1). The THI-box sequence is underlined. The start codon of *tbpA* is displayed at larger size as GTG, where the first G is considered +1 in the numbering system.

sYJ5, sYJ20 (SroA) and sYJ118 are all highly conserved within the different members of Enterobacteriaceae, although the coding sequences of sYJ5, sYJ20 and sYJ118 are also found in other families of bacteria (such as sYJ5 and sYJ118 in *Prevotella ruminicola*, sYJ20 in *Marinobacter aquaeolei* VT8), in plants (such as sYJ20 and sYJ118 in *Zea mays* cultivar line T63) and in animals (sYJ118 in *Gryllus bimaculatus*)*.* In contrast, sYJ75 is only found in *Salmonella*, *Enterobacter*, *Photorhabdus* and *Citrobacter*.

### sYJ20 (SroA), sYJ5, sYJ75 and sYJ118 in other species and relevance to other drug classes

We proceeded to determine whether the increased expression of these sRNAs would be *Salmonella* specific or drug-class specific. Hence, we assessed the levels of our sRNA candidates (sYJ5, sYJ20 and sYJ118) in other members of Enterobacteriaceae (*Klebsiella pneumoniae* and *Escherichia coli*) when challenged with sub-inhibitory levels of tigecycline (sYJ75 was not included since it is not encoded in the tested species). Additionally, in order to determine whether these sRNAs are upregulated solely as a result of tigecycline challenge or are generally upregulated as a result of sub-inhibitory antibiotic challenge, *S.* Typhimurium SL1344 was challenged with either half the MIC of ampicillin (1 μg/ml) or ciprofloxacin (0.0156 μg/ml). As shown in Figure 3B, none of the four tested sRNAs were upregulated in response to ciprofloxacin exposure, whilst three (sYJ5, sYJ75 and sYJ118) were found to be upregulated in the presence of ampicillin. Interestingly, *E. coli* did not upregulate the expression of the three candidate sRNAs (sYJ5, sYJ20 and sYJ118) in response to challenge at half the MIC of tigecycline. However, sYJ118 exhibited an elevated level of expression in *K. pneumoniae* in the presence of tigecycline (Figure 3B). Of note, although the sYJ20 (SroA) coding sequence is present in *K. pneumoniae*, two transcripts were detected after hybridisation. However it was the larger RNA species that appeared upregulated in RNA derived from *Klebsiella* cells challenged with half the MIC of tigecycline. Hence we surmise that this larger RNA transcript, consistent with the larger intergenic region in *K. pneumoniae*, is where the sYJ20 homolog coding sequence is located. From these results we show that the upregulation of sRNAs identified in this study are neither species nor drug specific in the presence of unrelated classes of antibiotics.

### 5’ Rapid Amplifed cDNA Ends (5’ RACE) of sYJ20 (SroA)

To determine the transcriptional start site (TSS) of sYJ20 (shared with the one of *tbpA*), we performed 5’ RACE analysis. As shown in Figure 5, the 5’ RACE result reveals that the TSS of sYJ20 and *tbpA* lies 129 bases upstream of the start codon of *tbpA*, consistent with previous findings [34].

### Quantitative real time PCR (qPCR)

**Figure 6:**
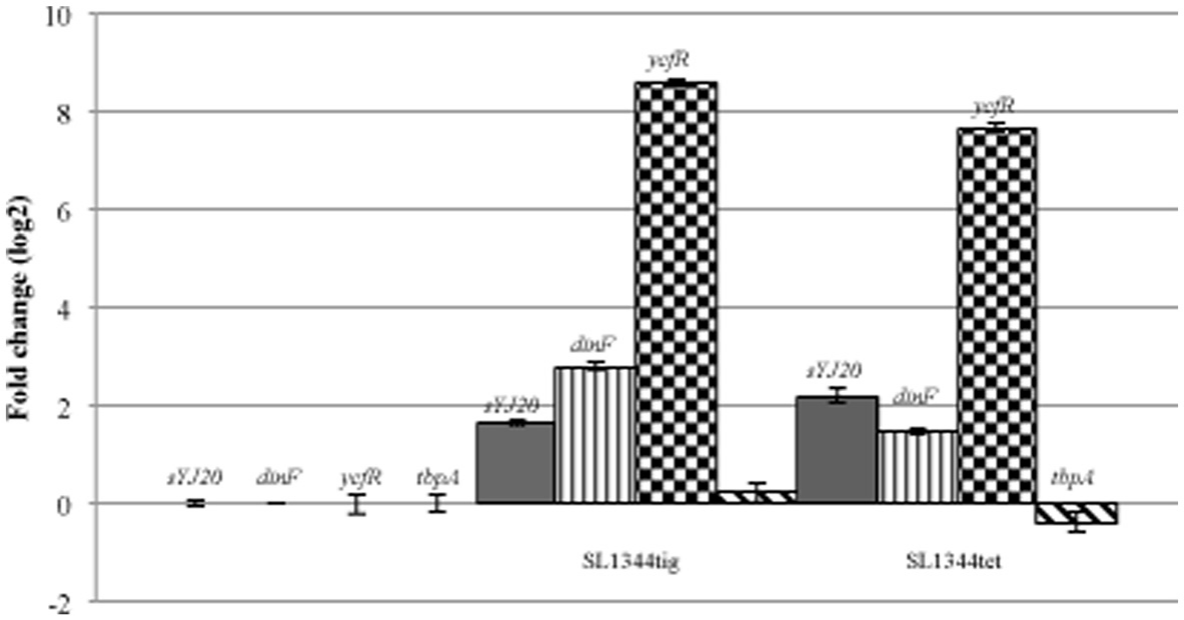
**qPCR on sYJ20, *tbpA *and stress responsive genes (*dinF *and *ycfR*) on SL1344 control (no challenge with antibiotics), SL1344 challenged with half the MIC of tigecycline (0.125 μg/ml), and SL1344 challenged with half the MIC of tetracycline (1 μg/ml).** QPCR was performed as described in Materials and Methods. All the fold changes are calculated relative to the value of the control (SL1344, unchallenged). Error bars are generated from at least 4 experiments.

sYJ20 (SroA): the upregulation of sYJ20 in *S.* Typhimurium challenged by half the MIC of tigecycline or tetracycline was quantified with qPCR. As shown in Figure 6, compared to the control, cells challenged by tigecycline or tetracycline produced ~3 fold more sYJ20. Interestingly, the transcription level of the downstream gene, *tbpA*, was hardly affected by the presence of the antibiotics. This suggests that sYJ20, but not the *tbpA* gene product, is upregulated as a result of tigecycline or tetracycline challenge.

*dinF* (encoding an efflux pump) and *ycfR* (encoding a putative outer membrane protein): as mentioned previously, the RNA transcripts of these two stress responsive genes were picked up in the sRNA cloning and is suggestive that half the MIC of tigecycline does induce a stress response in *S.* Typhimurium. In order to confirm this, we performed a qPCR on *S.* Typhimurium challenged by half the MIC of tigecycline or tetracycline, and compared the transcriptional levels of *dinF* and *ycfR* to the control. As shown in Figure 6, the transcriptional level of *dinF* increased to 7.0 and 2.8 fold when the cells were challenged by half the MIC of tigecycline and tetracycline, respectively; the level of *ycfR* increased to 390 and 210 fold when the cells were challenged by half the MIC of tigecycline and tetracycline, respectively.

### Survival rate assays

Survival rate assays were performed to investigate whether the deletion of sYJ20 (SroA) would highlight any phenotypic deficiencies when challenged with tigecycline. Our initial tests showed that the MICs of the mutant (YJ104) and the wild type strains (SL1344) were identical to tigecycline (MIC: 0.25 μg/ml in RDM). We then performed growth curves in RDM, where both SL1344 and YJ104 exhibited similar growth rates, as determined by OD_600_ readings, even in the presence of tigecycline (data not shown).

**Figure 7:**
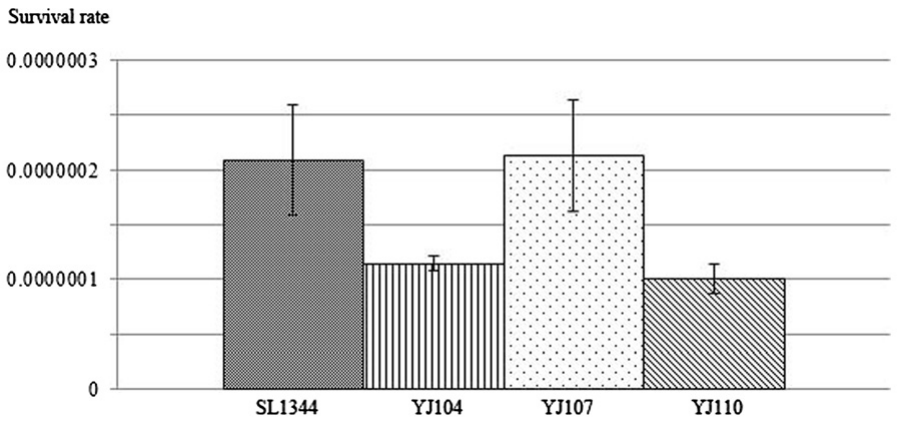
**Survival rate assays of SL1344, YJ104, YJ107 and YJ110 when cells were challenged with MIC of tigecycline.** Fresh overnight culture was spread on RDM plates either supplemented with MIC of tigecycline (0.25 μg/ml) or nothing (as a control). Colony number was determined after overnight incubation at 37°C. Survival rate was calculated as follows: cfu/ml on the tigecycline plate divided by cfu/ml on the control plate. *P* values were also calculated from at least three biological replicates. We found that statistical comparisons of SL1344 versus YJ104 (Δ*sYJ20*) and YJ107 (YJ104/pACYC177·*sYJ20*) versus YJ110 (YJ104/pACYC177) are significant (*P* < 0.05)

To determine whether sYJ20 confers an advantage to bacterial survival in the presence of tigecycline challenge, the survival frequencies were determined for the wild type SL1344 and YJ104 in the presence of 1 ×, 2 ×, 4 × and 8 × MIC of tigecycline. Both SL1344 and YJ104 failed to form any colonies on 2 ×, 4 × and 8 × MIC plates after overnight incubation at 37°C. The survival rates for SL1344 and YJ104 at 1 × the MIC were ~2.1 × 10^-7^ and 1.1 × 10^-7^ respectively (Figure 7). Despite this modest decrease, statistical analysis on four biological replicate experiments supports that the reduced survival rate observed in YJ104 is indeed significant (*P* < 0.05). The survival rate was restored upon complementation where YJ107 (YJ104/pACYC177·*sYJ20*) yielded a survival frequency close but higher than SL1344 (2.1 × 10^-7^, Figure 7), and as expected the plasmid control YJ110 (YJ104/pACYC177) had a similar survival rate to YJ104 (1.0 × 10^-7^, Figure 7). This reduction in the survival rate of YJ110 compared to the one of YJ107 was also found to be statistically significant (*P* < 0.05). Overall, it suggests that the absence of sYJ20 could confer a subtle but reduced survival rate in the presence of tigecycline.

## Discussion

Small RNAs are regulatory molecules that enhance a bacterium’s adaptability in a constantly changing environment [1-4]. As regulatory molecules, sRNAs have several advantages over their protein counterparts. Firstly, sRNAs consist of a short nucleotide sequence which does not require translation into a peptide sequence. This ensures that the response from sRNA mediated regulators would be much more rapid than protein mediated factors [35]. Accordingly, modelling studies suggest that due to the rapid kinetics associated with sRNA production, the downstream regulon response is correspondingly prompt when compared to protein based factors, a valuable trait in constantly evolving environments [35]. Moreover, base pairing flexibility presumably allows rapid evolution of sRNAs [35]. Finally, sRNA-mRNA interaction generally lacks specificity and often imperfect binding occurs ensuring that more than one target mRNA is affected, thereby expanding the repertoire of the sRNA regulators [8]. At antibiotic concentrations at or higher than the MIC, cells are likely to halt cellular replication and / or stop growing, or result in the accumulation of advantageous genomic mutations [36], which may permanently alter the transcriptional profiles of bacteria [37]. Hence we surmised that the sRNAs upregulated in the cells under these conditions may not be a direct result of antibiotic stress response but possibly due to genetic mutations or global perturbations. Therefore, a cDNA library was constructed from the cells that were challenged by half the MIC of tigecycline at mid-log phase.

In support of our hypothesis, our screen identified genes involved in the stress response when the bacterial cells were challenged with half the MIC of tigecycline. These include a SOS response gene, *dinF*, encoding a MATE family efflux pump, and a gene homologous to *ycfR* in *E. coli*, encoding a putative outer membrane protein. QPCR confirms the upregulation of the two genes when *S.* Typhimurium is challenged with half the MIC of tigecycline or tetracycline (Figure 6). Our finding of four sRNAs (sYJ20 (SroA), sYJ5, sYJ75 and sYJ118) that are upregulated in the presence of tigecycline or tetracycline provides the first direct evidence that sRNAs are differentially expressed upon antibiotic exposure. It is known that tetracycline triggers mRNA accumulation in bacteria [38]. However, this is unlikely to be the case as increased transcription was not noted for e.g. *tbpA* (open reading frame lying downstream of sYJ20, Figure 6), and the gene encoding the 5S RNA (Figure 4A).

Two of the four sRNAs (sYJ5 and sYJ75) we describe in this study are novel. Additionally, our work shows that these four sRNAs are not species specific as both sYJ20 and sYJ118 are upregulated in *K. pneumoniae* when challenged with half the MIC of tigecycline, or drug specific as sYJ5, sYJ75 and sYJ118 are upregulated as a result of ampicillin challenge (Figure 3B). Both sYJ118, previously identified as StyR-44 in *Salmonella*[39], and sYJ5, a novel sRNA discovered in this study, are located between 16S and 23S rRNA coding sequences (Figure 2C).

Both tigecycline and tetracycline target the 30S ribosomal subunit in bacterial cells. This might trigger over-production of the 16S-23S rRNA molecules, which also includes sYJ5 and sYJ118. This may raise the possibility that sYJ5 and sYJ118 are “by-products” rather than *bona fide* sRNA regulators. However, in support of sYJ5 and sYJ118 being classed as sRNAs, not all 16S-23S rRNA intergenic regions identified in our screen were upregulated in the presence of tigecycline when assessed by northern blots (data not shown). Furthermore, only sYJ118, not sYJ5, was upregulated in *K. pneumoniae* when challenged with tigecycline (Figure 3B). Taken together these data lead to the observation that these different inter 16S-23S rRNA regions (including the regions encoding either sYJ5 or sYJ118) may have alternative functions independent of rRNA processing, which could be regulatory sRNA.

In this work we have used the 5S RNA as a loading control for northern blot assays. Given that it is a ribosomal RNA we wondered whether the 5S RNA levels would be affected by either tigecycline or tetracycline exposure. As shown in Figure 4A, the 5S RNA expression levels were unaltered when the cells were challenged with half the MIC of tigecycline or tetracycline, and therefore it is a suitable loading control for the northern blot assays.

The four sRNAs (sYJ5, sYJ20, sYJ75 and sYJ118) that were upregulated as a response to tigecycline challenge in *S.* Typhimurium were also upregulated in tetracycline challenged cells (Figures 2A and 3A). This is not surprising since both tigecycline and tetracycline target the 30S ribosomal subunit. It is possible that the similar mechanisms of action of tetracycline and tigecycline trigger comparable stress-responsive pathways, which possibly include sYJ5, sYJ20, sYJ75 and sYJ118.

sYJ75 has not been previously described and thus is also a novel sRNA discovered in this study. Its conservation among several species and its upregulation in *S.* Typhimurium upon challenge with tigecycline and tetracycline, (Figures 2A, 3A) suggest that sYJ75, combined with its conservation across different species, may represent a common denominator in the response to tigecycline / tetracycline exposure. Interestingly, none of the four sRNAs were found upregulated when *S.* Typhimurium was exposed to ciprofloxacin, or when *E. coli* was challenged with tigecycline (Figure 3B).

When challenged with tigecycline, both *S.* Typhimurium and *K. pneumoniae* upregulated two sRNAs, namely sYJ20 and sYJ118 (Figure 3B). Despite encoding these sequences, no upregulation was noted in *E. coli* cells exposed to tigecycline compared to the unexposed controls (Figure 3B). This suggests two possibilities: the first, where the tigecycline stress response involving sRNAs in *E. coli* is different from that in *K. pneumoniae* and *S.* Typhimurium, and the second, where the sRNAs (sYJ20 and sYJ118) may be linked to regulatory networks contributing to tigecycline resistance, i.e. RamA, only found in *S.* Typhimurium and *K.pneumoniae* but not in *E. coli*[40,41]. However TargetRNA [42] predictions for sYJ20 for cognate mRNA binding partners, using default parameters, yields four mRNA sequences (Table 1). Of note, *pspB* and *pspA* which are involved in stress-response and the virulence attributes of several bacterial species [43] are potential targets of sYJ20. sYJ20-mediated control of the *psp* operon may explain the reduced fitness of the *sroA* (sYJ20) deleted *Salmonella* strain in a mouse infection model [44].

**Table 1:** TargetRNA predictions for sYJ20

**Rank**	**Gene**	**Synonym**	**Score**	**p-value**	**sRNA start**	**sRNA stop**	**mRNA start**	**mRNA stop**
1	pspB	STM1689	−60	0.00598756	17	28	9	−3
2	nrdI	STM2806	−60	0.00598756	17	28	9	−3
3	STM0269	STM0269	−59	0.00721216	7	29	16	−4
4	pspA	STM1690	−59	0.00721216	35	60	14	−10

The sequence of sYJ20 (as shown in Figure 5, bold letters) was applied as the input for TargetRNA ( http://snowwhite.wellesley.edu/targetRNA/) prediction with default parameters.

A recent study undertaken to map sRNA profiles in SL1344 using massive parallel sequencing technology identified 140 sRNAs. Notably, sYJ5 and sYJ75 were not identified in this large scale study which suggests that firstly, these sRNAs are produced as a result of conditional exposure e.g. tigecycline and secondly that our small scale screen is able to uncover novel sRNAs [34]. The encoding sequences of three sRNAs (sYJ5, sYJ75 and sYJ118) identified in this screen have more than one paralog within *S.* Typhimurium’s genome, making it difficult to pinpoint their exact roles in the bacterial response against antibiotic challenge through genetic analysis. Due to this reason, only sYJ20 and its associated phenotype were investigated further.

sYJ20, also known as SroA [5], is encoded immediately upstream of the *tbpAyabKyabJ* operon (homologous to *thiBPQ* in *E. coli*) and contains a THI-box sequence required as a riboswitch for the modulation of the *tbpAyabKyabJ* operon (Figure 5). The deletion of the chromosomal sequence of sYJ20 would have very likely removed the TSS of the downstream gene *tbpA* (Figure 5). However, *tbpA* transcript levels remained unaltered upon tigecycline / tetracycline exposure (Figure 6). Therefore the polar effect of the sYJ20 deletion is considered to be minimal.

When survival rate assays were performed a subtle but reproducible deficiency (*P* < 0.05) as reflected by a reduction in the viability in the Δ*sYJ20* strain (YJ104) compared to the wild type strain (SL1344) (Figure 7) was observed. This deficiency was alleviated when a plasmid encoding allele of sYJ20 was transformed in YJ104 (i.e. YJ107), where the vector only control (i.e. YJ110) did not (Figure 7). This subtle change of phenotype is not entirely surprising, as it has been observed that sRNA deletions usually have little, if any, effect [45]. In fact, sYJ20, or SroA, has been linked to other phenotypes such as reduced fitness by a Δ*sroA S.* Typhimurium strain (*sroA* encodes sYJ20) during competitive infection with the wild type strain in mice [44]. However it is not evident from the work whether the reduction in competitiveness of the Δ*sroA S.* Typhimurium strain is due to altered *tbpA* expression.

Previous work suggests that sYJ20 (SroA) may function as a riboswitch for the *tbpAyabKyabJ* (*thiBPQ*) operon [5] in *E. coli* and that this regulatory role does not require Hfq [46]. In our studies, we can show that the wild type strain *S.* Typhimurium (SL1344) produces sYJ20 (transcript size around 100 nts) in the presence of sub-inhibitory concentration of ciprofloxacin (0.0078 μg/ml) whilst the Δ*hfq* strain [7] produced less (Figure 4B). This suggests that sYJ20, apart from its putative riboswitch role, can act as a *trans*-regulatory sRNA, as Hfq is typically required for functionality and stability by *trans*-encoded sRNAs [47]. This is further supported by the two facts that A) the mild defect due to the chromosomal deletion of sYJ20 in SL1344 can be complemented by the plasmid-coding allele (YJ107), which cannot be attributed to its role as riboswitch, since the RNA transcripts of sYJ20 (on plasmid) and *tbpA* (on chromosome) are on separate strands, and B) sYJ20 was upregulated in *S.* Typhimurium challenged with half the MIC of tigecycline or tetracycline, where the transcriptional level of *tbpA* remained the same (Figure 6). The transcript size of sYJ20, as detected by northern blot analysis, is approximately 100 nts which is consistent with the size reported in *E. coli* (93 nts) [5]. As has been suggested previously, it is possible that sYJ20 is generated by transcription attenuation of *tbpAyabKyabJ*[5]; and the released short sYJ20 (around 100 nts) functions as a sRNA by regulating alternative targets *in trans* in the cell.

## Conclusions

Our work shows that sRNAs upregulated in response to tigecycline exposure can also be produced in a non drug or species specific manner. The deletion of the sRNA, sYJ20 (SroA) confers a subtle survival disadvantage in the presence of tigecycline, possibly due to its role as a *trans*-regulatory sRNA after tigecycline exposure. Our results although preliminary, suggest that sRNA levels can be altered upon antibiotic exposure and presumably provide an initial survival advantage under antibiotic challenge. However, ongoing analyses are required to dissect the regulatory impact(s) of sRNA upregulation and its contribution to antibiotic resistance in bacteria.

## Methods

### Growth conditions

Bacteria were cultured in Rich Defined Medium (RDM: 1 × M9 salts, 0.4% glucose, 1 × Essential Amino Acids (Gibco), 1 × Nonessential Amino Acids (Sigma-Aldrich, UK), 2 mM MgCl_2_, 0.1 mM CaCl_2_) unless otherwise stated. Typically, a strain was grown on a Luria-Bertani (LB) plate from frozen stock prior to experimental manipulations. A 1 in 100 dilution of fresh overnight culture was made in RDM and incubated in a 37°C shaker until OD_600_ reached 0.6, at which point half the MIC of the selected antibiotic (For SL1344: tigecycline (MIC = 0.25 μg/ml), tetracycline (MIC = 2 μg/ml), ciprofloxacin (MIC = 0.0312 μg/ml), or ampicillin (MIC = 2 μg/ml), for *K. pneumoniae*: tigecycline (MIC = 0.25 μg/ml), for *E. coli*: tigecycline (MIC = 0.0625 μg/ml), for JVS-0255: ciprofloxacin (MIC = 0.0156 μg/ml)) was added to the medium. The same volume of sterile water was added to another sample as a control. All strains used in this study are shown in Table 2.

**Table 2:** Strains and plasmids used in this work

**Strain**	**Genotype**	**Comment**
SL1344	*Salmonella enterica* serovar Typhimurium wild type	[48]
JVS-0255	SL1344 Δ*hfq*	[7]
MG1655	*Escherichia coli* wild type	[49]
Ecl8	*Klebsiella pneumoniae* wild type	[50]
YJ104	SL1344 ΔsYJ20*::*cat*	This work, derived from SL1344
YJ107	SL1344 Δ*sYJ20*::*cat* pYJ104	This work, derived from YJ104
YJ110	SL1344 Δ*sYJ20*::*cat* pACYC177	This work, derived from YJ104
**Plasmid**	**Genotype**	
pACYC177		
pYJ104	pACYC177 ·*sYJ20*	HindIII/BamHI fragment from PCR for SL1344 using primers sYJ20_HF and sYJ20_BR

*: *sYJ20* is the coding sequence for SroA.

### Minimum inhibitory concentration (MIC) determination

The MICs of all relevant strains in RDM to tigecycline, (gift from Wyeth Pharmaceuticals, US), tetracycline (Sigma-Aldrich, UK), ciprofloxacin and ampicillin (Sigma-Aldrich, UK) were determined and interpreted according to the BSAC protocols [51]. In order to check whether concentrations at half the MIC would induce stress response rather than kill the cells in liquid medium, half of the MIC of the antibiotic was added to liquid culture at OD_600_ = 0.6 (sterilised water was added to the control). After growth for an hour or overnight, an aliquot of the culture was taken and spread on plates, to determine colony forming unit per ml (cfu/ml). Additionally growth curves were also generated based on the OD_600_ readings. The stress response was confirmed by comparison of the antibiotic challenged cells to the control on both the growth curves and the cfu/ml.

### RNA extraction

Cells were grown to OD_600_ = 0.6 prior to the addition of the antibiotic. After 1 hour of exposure, cells were harvested by centrifugation. The cell pellet was then resuspended in TRIzol reagent (Invitrogen) and the total RNA was extracted according to Santhakumar *et al.*[52]. The resulting pellet was washed and resuspended in an appropriate amount of DEPC (Sigma, UK) treated water.

### cDNA library construction

The cDNA library was constructed (according to the manufacturer’s instruction) using the Exact START Small RNA Cloning kit from Epicentre (Cambio, UK). Briefly, total RNA was digested with DNase I to remove any contaminating DNA, and small RNAs were enriched with Epicentre enrichment solution by precipitating RNA molecules longer than 200 nts. The enriched RNAs were treated with phosphatase (Cambio, UK) to convert 5’ triphosphate group of RNA molecules to 5’ monophosphate, and a poly-A tail was added to the 3’ end (according to the manufacturer’s instruction). The 5’ end of RNA was ligated with Acceptor Oligo that carries a NotI restriction site. Reverse transcription was performed to yield first cDNA strand, using a primer with poly-T at its 3’ end to cover the poly-A tail of RNA samples, and an AscI restriction site. After RNase digestion, the sample was subject to a PCR with Small RNA PCR Primer 1 and 2. The product was digested by NotI and AscI (New England Biolabs) and was subsequently cloned into the cloning-ready pCDC1-K vector (Cambio, UK). Since the 5’ ligation adaptor differs from the 3’ ligation adaptor, the cloning of these putative small RNA molecules is directional. All oligonucleotides used in this study are listed in Table 3.

**Table 3:** Oligos used in this work

**Name**	**Sequence 5’-3’**	**Ref**
**For deletion of sY20 in SL1344**		
sYJ20_Cm_F	CTTGATTGCTGCCCGGCAACAAAA TCACTACACTAACGCCGTGTAGGC TGGAGCTGCTTC	This work
sYJ20_Cm_R	CTTTGCACCTCAGTTAAAGAGTGG CAAAGGACTTGAGATGGGAATTAG CCATGGTCC	
**For cloning sYJ20 coding sequence onto pACYC177**		
sYJ20_HF	CCCAAGCTTCTTGATTGCTGCCCGG CAACAA	This work
sYJ20_BR	CGGGATCCCTTGAGAGGTAGCCTC AAATCCCTT	
**For the northern blot assays**		
NsYJ5	AGGTTTTACTGCTCGTTTTTCA	This work
NsYJ20	ATCCGGATCAGGTTCGACGGGTAT	
NsYJ75	GCGGGGATTTCTTCCCTTGC	
NsYJ118	TGTGTTTCAATTTTCAGCTTGATCCAGATT	
5S	CTACGGCGTTTCACTTCTGAGTTC	[53]
**For qPCR**		
16SF	GTTACCCGCAGAAGAAGCAC	This work
16SR	CTACGCATTTCACCGCTACA	
tbpAF	GGCTGGAAAAACGACACATT	
tbpAR	TAGACTTTGCGCATCCACAG	
ycfR-F	TGCCGTACTGAGTTCGCTCT	
ycfR-R	GGGCCGGTAACAGAGGTAAT	
dinF-F	TTACTGGGGCTGGTCGATAC	
dinF-R	GCCAGCAATAACGGTTGAAT	
q5S-F	CATGCCGAACTCAGAAGTGA	
q5S-R	AGTTCCCTACTCTCGCATGG	
**For 5’RACE**		
GSP1	GGCGAAATAGCCGTAAT	This work
GSP2	GGGCACCTTGACCGCTTCAT	
GSP3	GCCACGCCGCTTTTGGCAAA	

### Northern blots

Ten micrograms of total RNA was separated on 8% polyacrylamide gel containing 8 M urea, and electro-transferred onto Hybond-N nylon membrane (GE Healthcare). The membrane was hybridised and washed according to Vogel *et al.*[54], and exposed to a phosphor-imager (Fuji). Relative levels of increase in expression were determined by Multi Gauge 2.2 (Fujifilm). The bands were first normalised to the 5S RNA levels prior to calculating the fold increase of challenged versus unchallenged cells. The oligonucleotide probes used in the northern blot experiments are listed in Table 3, and were end-labelled with γ^32^P-ATP using T4-polynucleotide kinase and purified prior to blot hybridisation.

### Chromosomal sYJ20 (SroA) inactivation

The chromosomal inactivation of sYJ20 (SroA) was performed according to the manipulation strategy outlined by Datsenko and Wanner [55]. Briefly, primers (sYJ20_Cm_F and sYJ20_Cm_R, sequences listed in Table 3) with ~40 bases with 5’ end homology to the flanking regions of the sYJ20 coding sequence were used to amplify the *cat* locus on pKD3 by PCR. The PCR product was transformed into *S.* Typhimurium SL1344 carrying the plasmid pKD46. The transformed cells were selected on LB plates supplemented with chloramphenicol. Colonies were picked after an overnight incubation and the replacement of the chromosomal sYJ20 coding sequence with the *cat* cassette was verified by PCR and sequencing.

### Quantitative Real Time PCR (qPCR)

All the primers for qPCR were tested for amplification efficiencies prior to use. cDNA was made with SuperScript® VILO^TM^ cDNA Synthesis Kit (Invitrogen), which was then subject to qPCR with Platinum® SYBR® Green qPCR SuperMix-UDG (Invitrogen). The qPCR was performed using the Mx3005P qPCR system (Agilent/Strategene). Analyses of the QPCR data were undertaken using the MxPro algorithms (Agilent, UK) where the normalisation of the amplification data was to the 5S RNA levels.

### Complementation assay

The sequence spanning 40 bases upstream and 6 bases downstream up to the sYJ20 sRNA encoding sequence was amplified with primers sYJ20-HF and sYJ20-BR and cloned into pACYC177. The recombinant plasmid carrying the sYJ20 encoding sequence was verified by sequencing before transformation into YJ104 (SL1344 Δ*sYJ20*) to yield YJ107. Empty pACYC177 was also transformed into YJ104 to yield YJ110, used as a negative control. The levels of sY20 expression were confirmed by northern blots.

### 5’ RACE

In order to determine the TSS of sYJ20 and *tbpA*, we employed the 5’ RACE System for Rapid Amplification of cDNA Ends (version 2.0, Invitrogen). Briefly, the first strand cDNA was produced using SuperScript^TM^ II Reverse Transcriptase (Invitrogen) with the GSP1 primer specifically matching to the *tbpA* RNA transcript. Following purification with the S.N.A.P column (Invitrogen), the 5’ end of the first strand cDNA was tailed with multiple C (cytidines) with dCTP and TdT. A PCR was performed with the Abridged Anchor Primer (Invitrogen) that targets the dC-tailed 5’ cDNA end, and the GSP2 primer attaching to the RNA transcript upstream of the GSP1 matching region. A nested PCR was also performed to increase the specificity with the nested GSP3 primer and the AUAP primer (Invitrogen). The PCR product was ligated onto the pGEM-T EASY vector, and was sequenced with the T7 Forward primer or the SP6 Reverse primer.

### Survival rate assay

To assess the fitness of strains challenged with tigecycline, a survival rate assay of the wild type (SL1344), the Δ*sYJ20* mutant (YJ104), the plasmid complemented strain (YJ107), and the vector only control (YJ110) was performed. One hundred microlitres of cells from fresh overnight RDM cultures were spread evenly on RDM plates supplemented with tigecycline at the MIC, 2 × MIC, 4 × MIC or 8 × MIC. The same batch of cells was also spread on RDM plates with no antibiotics to establish the baseline levels.

## Authors’ contributions

TS and JY designed all the experiments. JY carried out the experiments. TS and JY wrote the manuscript. Both authors read and approved the final manuscript.
